# Supplementary material for: Targeted inhibition of BET proteins in HPV-16 associated head and neck squamous cell carcinoma reveals heterogeneous transcription response
Source: bioRxiv. 2023 Oct 4:2023.10.02.560587. Preprint. [Version 1] doi: 10.1101/2023.10.02.560587 (PMC10592929; doi:10.1101/2023.10.02.560587)
Supplement: Supplement 1 [file NIHPP2023.10.02.560587v1-supplement-1.pdf]

bioRxiv preprint doi: https://doi.org/10.1101/2023.10.02.560587; this version posted October 4, 2023. The copyright holder for this preprint (which was not certified by peer review) is the author/funder, who has granted bioRxiv a license to display the preprint in perpetuity. It is made available under aCC-BY 4.0 International license.

Supplementary figures

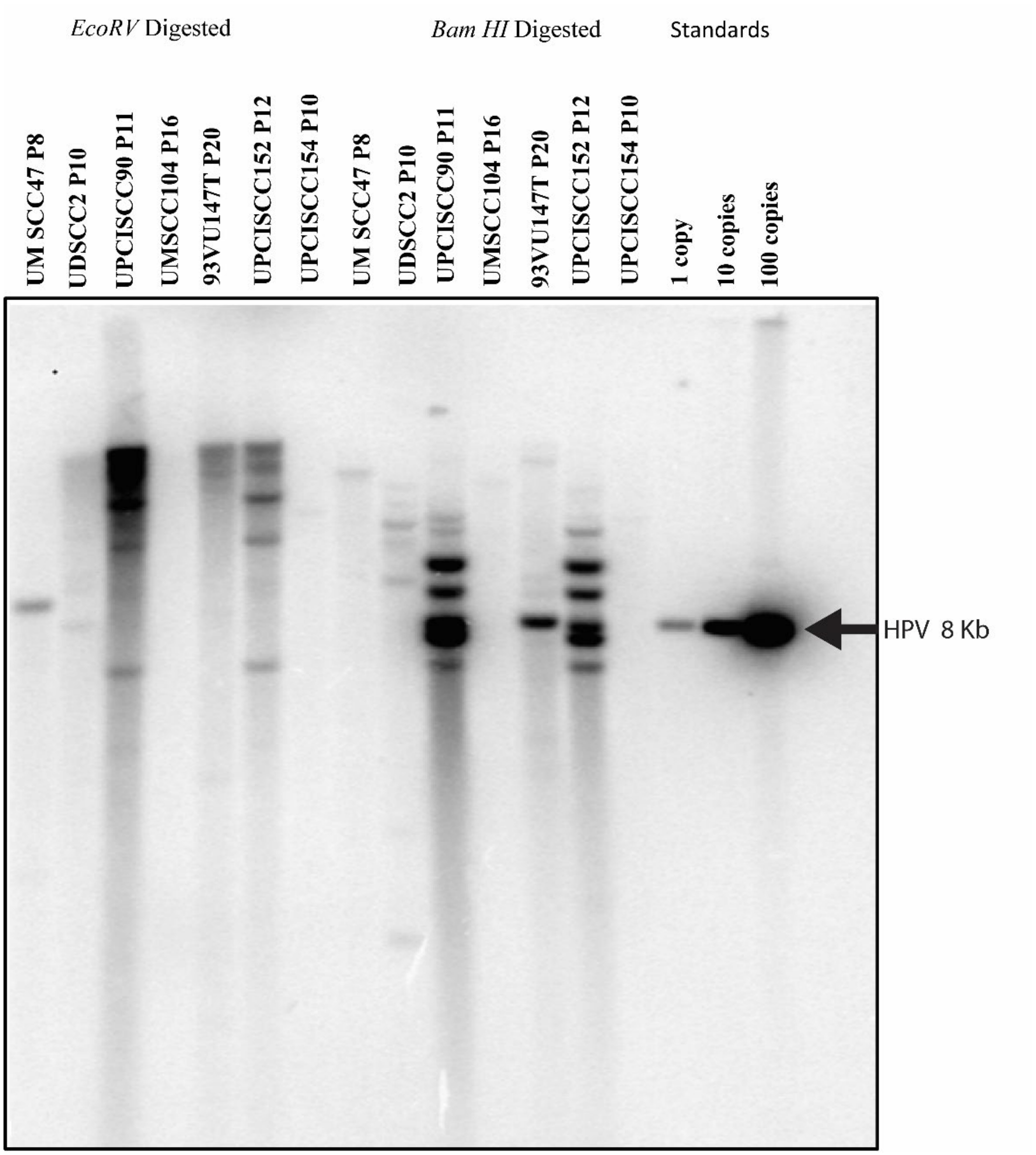

Figure.U S1. Southern blot hybridization of restriction digested of seven HPV cell lines. Control HPV ~ 8 kb plasmid with copy number of 1, 10 and 100 was loaded to validate the presence of HPV DNA in these cancer cell lines. P indicates passage number.

bioRxiv preprint doi: https://doi.org/10.1101/2023.10.02.560587; this version posted October 4, 2023. The copyright holder for this preprint (which was not certified by peer review) is the author/funder, who has granted bioRxiv a license to display the preprint in perpetuity. It is made available under aCC-BY 4.0 International license.

Nonlinear Curve Fit Dose Response Parameters

	Value	Standard Error	t-Value	Prob>|t|	Dependency
A1	42.49093	3.25572	13.05115	1.24718E-5	0.48754
A2	103.98268	13.99807	7.42836	3.06282E-4	0.84385
LOGx0	-7.18672	0.18486	-38.87645	1.93494E-8	0.83835
p	-1.66667	0.77999	-2.13678	0.07649	0.75364
span	61.49176	15.33673			
EC20	1.49458E-7	4.53401E-8			
EC50	6.50552E-8	2.76912E-8			
EC80	2.83169E-8	2.14424E-8			
A1	47.62577	2.86894	16.60048	3.04803E-6	0.43487
A2	98.32513	5.57133	17.64842	2.12477E-6	0.53333
LOGx0	-6.95525	0.12865	-54.06292	2.68886E-9	0.55615
p	-1.66667	0.65585	-2.54121	0.04401	0.53136
span	50.69936	6.73198			
EC20	2.54674E-7	8.61312E-8			
EC50	1.10853E-7	3.2838E-8			
EC80	4.82516E-8	2.53215E-8			
A1	52.08379	2.1801	23.89059	3.53199E-7	0.49136
A2	94.55697	4.12771	22.90787	4.5335E-7	0.61312
LOGx0	-6.79148	0.1189	-57.11942	1.93422E-9	0.5911
p	-1.66667	0.54071	-3.08235	0.0216	0.5907
span	42.47318	5.06964			
EC20	3.71323E-7	1.11008E-7			
EC50	1.61628E-7	4.42499E-8			
EC80	7.03525E-8	3.19445E-8			
A1	59.52425	1.43547	41.46682	1.31561E-8	0.28423
A2	97.63684	2.81272	34.71266	3.80814E-8	0.60298
LOGx0	-6.68143	0.08283	-80.66333	2.44453E-10	0.48042
p	-1.66667	0.47598	-3.50152	0.0128	0.4544
span	38.11259	3.35963			
EC20	4.78418E-7	1.28869E-7			
EC50	2.08244E-7	3.97174E-8			
EC80	9.06433E-8	3.04773E-8			
A1	16.85289	1.66196	10.14034	5.34922E-5	0.14153
A2	101.86837	15.80913	6.44364	6.61271E-4	0.69777
LOGx0	-7.0341	0.16566	-42.46106	1.14175E-8	0.75703
p	-1.66667	0.62101	-2.6838	0.03635	0.57388
span	85.01549	16.09625			
EC20	2.12393E-7	5.74289E-8			
EC50	9.24495E-8	3.52645E-8			
EC80	4.0241E-8	2.57667E-8			
A1	68.48517	4.95197	13.82989	8.8954E-6	0.40994
A2	109.4464	5.0427	21.70394	6.24656E-7	0.57057
LOGx0	-6.51796	0.28336	-23.00228	4.42407E-7	0.33373
p	-1.66667	1.578	-1.05619	0.33154	0.59263
span	40.96123	8.06861			
EC20	6.97064E-7	6.47238E-7			
EC50	3.03415E-7	1.97967E-7			
EC80	1.32069E-7	1.46452E-7			
A1	74.91165	8.45115	8.86407	1.14692E-4	0.80406
A2	107.50372	2.15514	49.88245	4.35383E-9	0.35344
LOGx0	-5.82047	0.22501	-25.86765	2.20081E-7	0.88767
p	-1.66667	1.24713	-1.3364	0.22986	0.77963
span	32.59208	9.01			
EC20	3.4735E-6	3.79727E-6			
EC50	1.51193E-6	7.83335E-7			
EC80	6.58104E-7	2.24638E-7			

Reduced Chi-sqr = 0.197218498215 COD(R^2) = 0.97049115006367
Iterations Performed = 12 Total Iterations in Session = 12
All datasets were fitted successfully.
Standard Error was scaled with square root of reduced Chi-Sqr. span, EC20, EC50, EC80 are derived parameter(s).

Statistics

bioRxiv preprint doi: https://doi.org/10.1101/2023.10.02.560587; this version posted October 4, 2023. The copyright holder for this preprint (which was not certified by peer review) is the author/funder, who has granted bioRxiv a license to display the preprint in perpetuity. It is made available under aCC-BY 4.0 International license.

	UM-SCC-47	UD-SCC-2	UPCI-SCC-90	93VU147T	UPCI-SCC-154	UM-SCC-104	UPCI-SCC-152
Number of Points	10	10	10	10	10	10	10
Degrees of Freedom	6	6	6	6	6	6	6
Reduced Chi-Sqr	0.69464	0.32555	0.31803	0.46139	1.30944	0.72104	0.19722
Residual Sum of Squares	4.16786	1.9533	1.90819	2.76832	7.85662	4.32623	1.18331
R-Square (COD)	0.96798	0.98163	0.95816	0.95835	0.98063	0.88452	0.97049
Adj. R-Square	0.95197	0.97245	0.93723	0.93753	0.97094	0.82678	0.95574
Fit Status	Succeeded(100)	Succeeded(100)	Succeeded(100)	Succeeded(100)	Succeeded(100)	Succeeded(100)	Succeeded(100)

Fit Status Code :
100 : Fit converged. Chi-Sqr tolerance value of 1E-9 was reached

		DF	Sum of Squares	Mean Square	F Value	Prob>F
UM-SCC-47	Regression	4	3505.71854	876.42963	1261.69645	6.69166E-9
	Residual	6	4.16786	0.69464		
	Uncorrected Total	10	3509.8864			
	Corrected Total	9	130.15589			
UD-SCC-2	Regression	4	1694.34777	423.58694	1301.14495	6.10211E-9
	Residual	6	1.9533	0.32555		
	Uncorrected Total	10	1696.30107			
	Corrected Total	9	106.33643			
UPCI-SCC-90	Regression	4	1764.8204	441.2051	1387.29699	5.03577E-9
	Residual	6	1.90819	0.31803		
	Uncorrected Total	10	1766.72859			
	Corrected Total	9	45.60214			
93VU147T	Regression	4	1525.05995	381.26499	826.34737	2.37625E-8
	Residual	6	2.76832	0.46139		
	Uncorrected Total	10	1527.82826			
	Corrected Total	9	66.4735			
UPCI-SCC-154	Regression	4	1350.58848	337.64712	257.85689	7.70464E-7
	Residual	6	7.85662	1.30944		
	Uncorrected Total	10	1358.4451			
	Corrected Total	9	405.51057			
UM-SCC-104	Regression	4	10685.41485	2671.35371	3704.87042	2.65067E-10
	Residual	6	4.32623	0.72104		
	Uncorrected Total	10	10689.74108			
	Corrected Total	9	37.4633			
UPCI-SCC-152	Regression	4	3210.35867	802.58967	4069.54558	2.0003E-10
	Residual	6	1.18331	0.19722		
	Uncorrected Total	10	3211.54198			
	Corrected Total	9	40.10021			

At the 0.05 level, the fitting function is significantly better than the function y=0.

bioRxiv preprint doi: https://doi.org/10.1101/2023.10.02.560587; this version posted October 4, 2023. The copyright holder for this preprint (which was not certified by peer review) is the author/funder, who has granted bioRxiv a license to display the preprint in perpetuity. It is made available under aCC-BY 4.0 International license.

Residual Plots

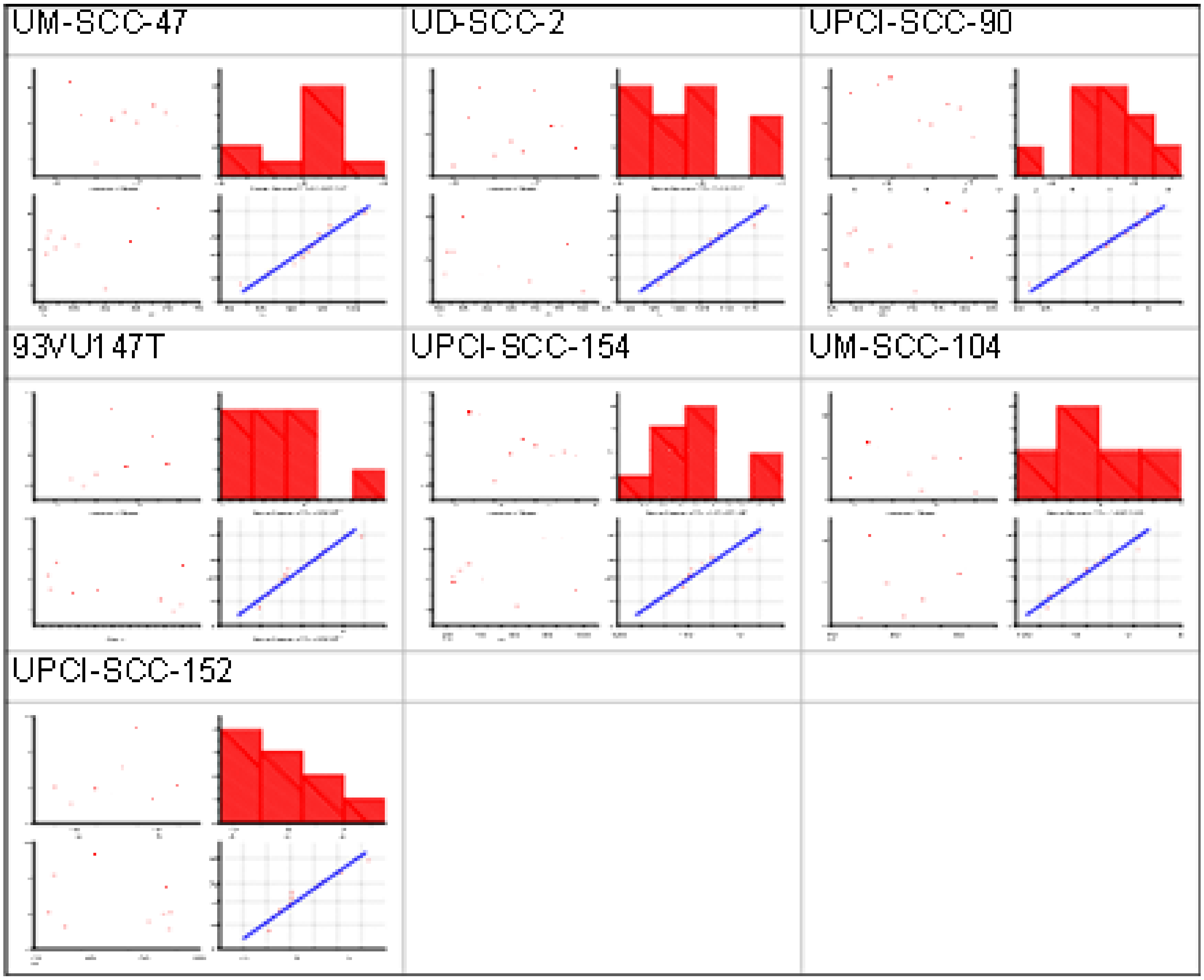

Figure. S2. The IC50 values for JQ1 treatments for all cell lines were extracted from applying the Nonlinear Curve Fit using the Levenberg Marquardt algorithm using the DoseResp model. The residual fits confirm that the IC50 values were fitted to the equation y = A1 + (A2-A1)/(1 + 10^((LOGx0-x)*p))

bioRxiv preprint doi: https://doi.org/10.1101/2023.10.02.560587; this version posted October 4, 2023. The copyright holder for this preprint (which was not certified by peer review) is the author/funder, who has granted bioRxiv a license to display the preprint in perpetuity. It is made available under aCC-BY 4.0 International license.

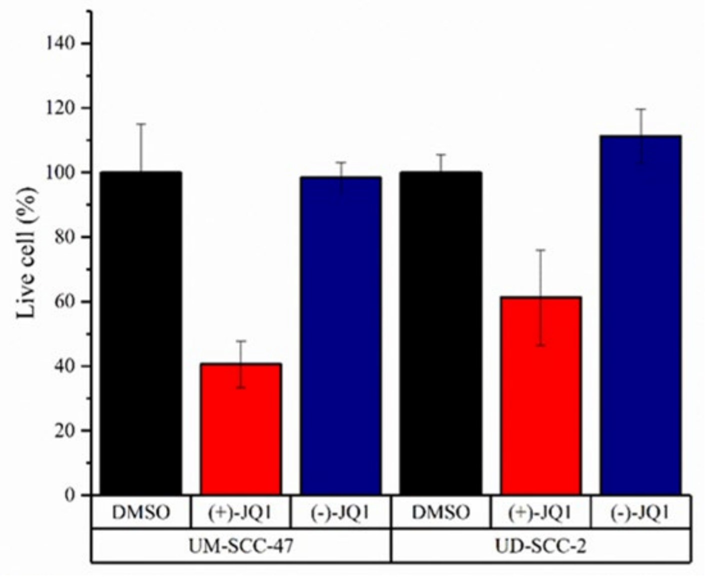

Figure. S3. HPV-positive (UM-SCC-47, UD-SCC-2) head and neck cancer cell lines were treated with DMSO (vehicle), 0.5 μM (+)-JQ1 or (-)-JQ1 for 96 hours. The number of live cells in each condition was counted using a TC20 Biorad cell counter and trypan blue exclusion. The cell numbers were then normalized to the DMSO-treated group. With (+)-JQ1 treatment for 96 hours, the number of live cells was significantly reduced compared to vehicle control (DMSO). While the group treated with (-)-JQ1, a stereoisomer of (+)-JQ1, had a percentage of live cells similar to the vehicle control group. This suggests (-)-JQ1 has minimal effect on the proliferation of head and neck cancer cells at the concentration used in this study.

bioRxiv preprint doi: https://doi.org/10.1101/2023.10.02.560587; this version posted October 4, 2023. The copyright holder for this preprint (which was not certified by peer review) is the author/funder, who has granted bioRxiv a license to display the preprint in perpetuity. It is made available under aCC-BY 4.0 International license.

A

Gene	UD:SCC2	p-value	UM:SCC47	p-value	UPCI:SCC90	p-value	UM:SCC104	p-value	93VU147T	p-value	UPCI:SCC152	p-value	UPCI:SCC154	p-value
E1	0	0	-2.633419	0.0142659	-2.0947516	0.19604723	-1.8805472	3.53E-30	-0.313392	0.535193	-0.5210918	0.0175069	-0.76563525	0.002787
E2	0.028157	0.925164	0.3078121	0	-1.702974	0.44015037	-2.2515172	3.14E-26	-0.934486	0.001518	-0.42040868	0.019755157	2.50569741	0
E5	0.082004	0.868666	-3.565674	0.0005241	-0.6181522	0	-1.4724131	0	2.238484	0	-2.61200436	0.196609045	0	0
E6	-0.19341	0.673064	-5.522024	2.85E-11	-3.2982762	0.03156495	-2.9374281	1.17E-40	-0.710889	0.002741	-0.63551982	0.002798126	-1.74772772	6.29E-08
E7	-0.18416	0.554812	-3.656829	2.07E-12	-3.2294627	0.05105591	-1.9517384	1.42E-05	-0.768849	0.1141	-1.68614212	1.20E-12	-1.38476642	0.003166
L1	-0.03963	0.911318	-0.279514	0.866561	-1.2206026	0.66441699	-2.6533874	0.02441571	0.248925	0.738637	0.579047522	0.057621392	0	0
L2	0.034996	0.955862	-2.58422	0.1557063	-1.0924648	0.75231265	-2.0429019	0.14720595	0.649898	0.348262	0.199274991	0.643661439	0	0

B

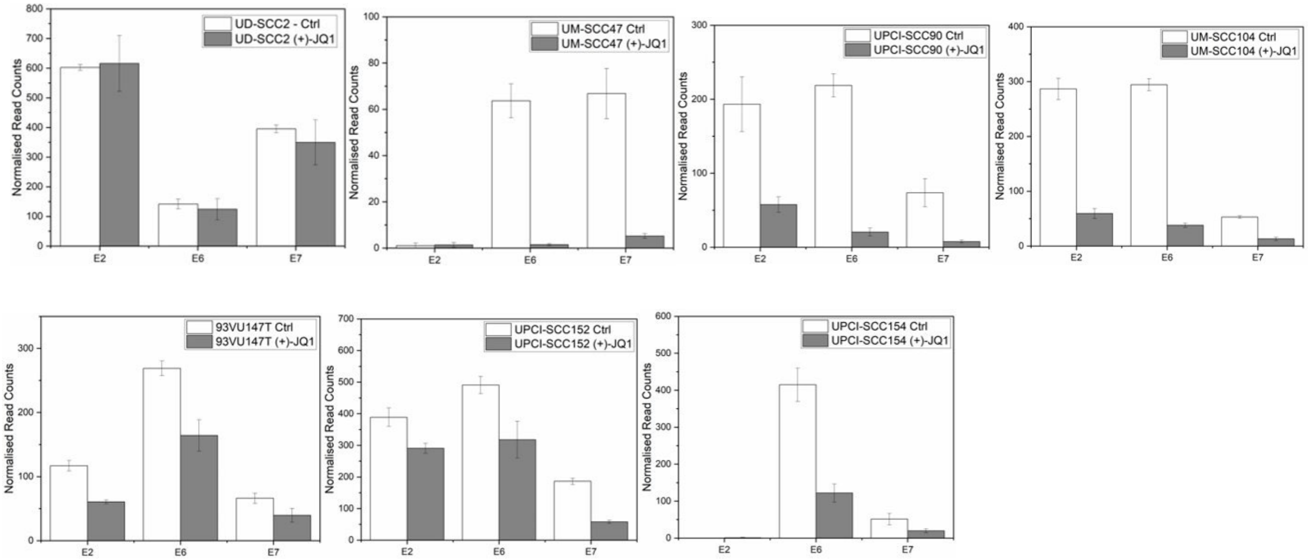

Figure S4. **A.** RNA-sequencing analyses of viral genes with log2fold change and its p-values. **B.** Normalized read counts of viral genes E2, E6, and E7 suggest the abundance of viral transcripts in seven HPV16-associated HNSCC cell lines.

bioRxiv preprint doi: https://doi.org/10.1101/2023.10.02.560587; this version posted October 4, 2023. The copyright holder for this preprint (which was not certified by peer review) is the author/funder, who has granted bioRxiv a license to display the preprint in perpetuity. It is made available under aCC-BY 4.0 International license.

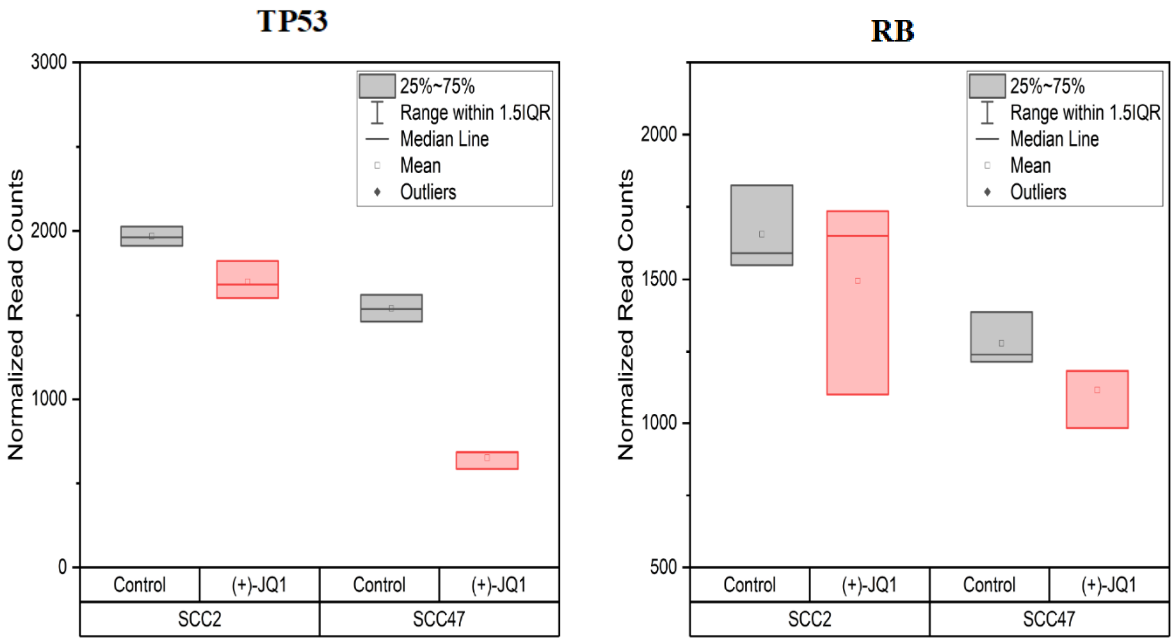

Figure S5. Box plot depicting normalized read counts of TP53 and RB from RNA-sequencing suggesting that despite viral E6 and E7 expression, both TP53 and RB RNA levels are abundantly expressed.

bioRxiv preprint doi: https://doi.org/10.1101/2023.10.02.560587; this version posted October 4, 2023. The copyright holder for this preprint (which was not certified by peer review) is the author/funder, who has granted bioRxiv a license to display the preprint in perpetuity. It is made available under aCC-BY 4.0 International license.
